# SARS-CoV-2 infection in farmed minks, associated zoonotic concerns, and importance of the One Health approach during the ongoing COVID-19 pandemic

**DOI:** 10.1080/01652176.2020.1867776

**Published:** 2021-01-18

**Authors:** Khan Sharun, Ruchi Tiwari, Senthilkumar Natesan, Kuldeep Dhama

**Affiliations:** a Division of Surgery, ICAR-Indian Veterinary Research Institute, Bareilly, Uttar Pradesh, India; b Department of Veterinary Microbiology and Immunology, College of Veterinary Sciences, Uttar Pradesh Pandit Deen Dayal Upadhyaya Pashu Chikitsa Vigyan Vishwavidyalaya Evam Go Anusandhan Sansthan (DUVASU), Mathura, Uttar Pradesh, India; c Department of Infectious Diseases, Indian Institute of Public Health Gandhinagar, Gandhinagar, Gujarat, India; d Division of Pathology, ICAR-Indian Veterinary Research Institute, Bareilly, Uttar Pradesh, India

**Keywords:** Neovison vison, farmed minks, COVID-19, SARS-CoV-2, zoonosis, pathogenesis, species barrier, cross species jumping

## Abstract

The coronavirus disease 2019 (COVID-19) pandemic has now affected over 72.5 million people worldwide, with nearly 1.6 million deaths reported globally as of December 17, 2020. SARS-CoV-2 has been implicated to have originated from bats and pangolins, and its intermediate animal hosts are being investigated. Crossing of the species barrier and exhibition of zoonosis have been reported in SARS-CoV-2 in farm (minks), domesticated (cats and dogs), and wild animals (tigers, puma, and lions). Recently, the rapid spread of SARS-CoV-2 infection was reported in mink farms, which led to the death of a myriad minks. The clinical and pathological findings of SARS-CoV-2 infection and the rapid animal-to-animal transmission in minks are almost similar to the findings observed in patients with COVID-19. Additionally, the rapid virus transmission among minks and the associated mutations resulted in a new mink-associated variant that was identified in both minks and humans, thereby providing evidence of mink-to-human transmission of SARS-CoV-2. The new mink-associated SARS-CoV-2 variant with a possible reduced sensitivity to neutralizing antibodies poses serious risks and is expected to have a direct effect on the diagnostic techniques, therapeutics, and vaccines that are currently under development. This article highlights the current evidence of SARS-CoV-2 infection in farmed minks, and provides an understanding of the pathogenesis of COVID-19 in minks and the associated zoonotic concerns of SARS-CoV-2 transmission from minks to humans with an emphasis on appropriate mitigation measures and on the necessity of adopting the One Health approach during the COVID-19 pandemic.

## 1. Introduction

Severe acute respiratory syndrome coronavirus 2 (SARS-CoV-2), the virus which causes coronavirus disease 2019 (COVID-19) in humans, was first reported in December 2019 (Malik et al. 2020). The SARS-CoV-2 that emerged in Wuhan, China, had a high genomic similarity with its predecessor, severe acute respiratory syndrome coronavirus (SARS-CoV), compared with the Middle East respiratory syndrome coronavirus (MERS-CoV) (Sharun et al. 2020a). COVID-19 received a pandemic status within a short period because of the high transmission potential of SARS-CoV-2 via respiratory droplets and human contacts (Dhama et al. 2020a).

SARS-CoV-2 is the third zoonotic coronavirus affecting human beings following the emergence of SARS-CoV in 2002 and MERS-CoV in 2012 (Wang and Eaton 2007; Hemida et al. 2017; Dhama et al. 2020a). Both SARS-CoV and MERS-CoV are believed to have been transmitted to humans through an intermediate mammalian host from a natural reservoir host (Wang and Eaton 2007; Hemida et al. 2017). Horseshoe bats of the genus *Rhinolophus* are considered the reservoir host of SARS-CoV while masked palm civets (*Paguma larvata*) act as the intermediate host (Wang and Eaton 2007). However, it was dromedary camels (*Camelus dromedarius*) that transmitted MERS-CoV to humans (Hemida et al. 2017). Similarly, SARS-CoV-2 is believed to have crossed the species barrier, possibly exhibiting transmission from the reservoir host, bats, and finally infecting humans through an intermediate species (Hobbs and Reid 2020; Malik et al. 2020; Dhama et al. 2020b). The intermediate host not only acts as the bridge that links the natural reservoir host and a susceptible population, but also acts as a host in which the virus undergoes gradual evolution (Zhao et al. 2020a). The entry of SARS-CoV-2 into the host cell is mediated through the interaction of the viral spike protein or the S protein with the host angiotensin-converting enzyme 2 (ACE2) receptor (Low-Gan et al. 2020).

SARS-CoV-2 has been implicated to have originated from bats and pangolins, while other animals have been investigated to identify any intermediate host. The recent detection of SARS-CoV-2 in farm (minks), domesticated (cats and dogs), and wild animals (tigers, puma, and lions) have increased concerns regarding the zoonotic sources of COVID-19 (Hobbs and Reid 2020; Jo et al. 2020; OIE 2020; Oreshkova et al. 2020; Sit et al. 2020; Tiwari et al. 2020; Mallapaty 2020a; Shi et al. 2020; Dhama et al. 2020b). Ferrets, cats, and primates have also been reported to be susceptible to experimental SARS-CoV-2 infection (Gollakner and Capua 2020; Shi et al. 2020). The increasing reports of SARS-CoV-2 in animal species indicate the need to understand and evaluate the susceptibility of animals as it is of utmost importance to public health and the economy. Furthermore, our understanding of the pathogenesis of SARS-CoV-2 will help to develop suitable animal models for therapeutic agent and vaccine development.

Among the animal species that are susceptible to natural SARS-CoV-2 infection, farmed minks pose a considerable threat to public health (Oreshkova et al. 2020; Oude Munnink et al. 2020). SARS-CoV-2 infection has been reported in both European (*Mustela vison*) and American (*Neovison vison*) minks (Manes et al. 2020). The rapid transmission of SARS-CoV-2 among minks and the associated mutations has resulted in a new mink-associated variant that has been identified in both minks and humans (Oude Munnink et al. 2020; WHO 2020a). In this article, we discuss the current evidence of SARS-CoV-2 infection in minks and its implications in understanding the pathogenesis of COVID-19.

## 2. SARS-CoV-2 crosses the species barrier

Experimental inoculation in animal species, such as cats (Shi et al. 2020), dogs (Shi et al. 2020), rhesus macaques (*Macaca mulatta*) (Deng et al. 2020), cynomolgus macaques (*Macaca fascicularis*) (Rockx et al. 2020), common marmosets (*Callithrix jacchus*) (Lu et al. 2020), African green monkey (*Chlorocebus aethiops*) (Woolsey et al. 2020), cattle (*Bos taurus*) (Ulrich et al. 2020), ferrets (*Mustela putorius*) (Schlottau et al. 2020; Shi et al. 2020), golden (Syrian) hamsters (*Mesocricetus auratus*) (Sia et al. 2020), rabbits (Mykytyn et al. 2020), tree shrew (*Tupaia belangeris*) (Zhao et al. 2020b), and fruit bats (*Rousettus aegyptiacus*) (Schlottau et al. 2020), suggest that these species are susceptible to SARS-CoV-2 infection. Among these susceptible species, cats, ferrets, and hamsters can further transmit the virus amongst the same species under experimental conditions (Shi et al. 2020; Sia et al. 2020). Even though not yet reported, these animals may further transmit the virus to humans, other animal species, or both. Although several species have exhibited susceptibility to SARS-CoV-2 under experimental conditions, the natural infection has only been reported as sporadic cases in cats, dogs, lions, puma, tigers, and farmed minks (Muñoz-Fontela et al. 2020; OIE 2020; Oreshkova et al. 2020; Tiwari et al. 2020; Sharun et al. 2020b).

SARS-CoV-2 possesses certain characteristic genomic features that enable inter-species transmission and adaptation to the conditions in a new host. Therefore, it is necessary to investigate the evolutionary dynamics of the viral genome and its impact on differential host selection (Ul-Rahman et al. 2020). Comparative analysis of ACE2 homologous proteins was performed to verify the conservation of specific amino acid residues. It has been observed that the peptide ‘353-KGDFR-357’ is the most conserved peptide located on the surface of the ACE2 and binds to the S protein receptor-binding domain (RBD), which is located on the surface of the ACE2 molecule (Hayashi et al. 2020a). Furthermore, sequence alignments have identified that the ACE2 proteins exhibited high homology and complete conservation of the five amino acid residues (353-KGDFR-357) among humans, cats, tigers, dogs, and minks (Hayashi et al. 2020a). Theoretically, SARS-CoV-2 may be transmitted from companion animals such as dogs and cats to humans and vice versa through droplets in the air, saliva, and bites of the infected animals. Other practices involving direct contact with pets, including snuggling, petting, and being kissed or licked may further increase the risk of SARS-CoV-2 transmission between the owners and their pets (Csiszar et al. 2020). However, the available data indicate that the risk of SARS-CoV-2 transmission from animals to humans is low (Salajegheh Tazerji et al. 2020). Therefore, even though transmission of SARS-CoV-2 from companion animals (cats and dogs) to humans has not been reported thus far, appropriate preventive measures should be considered while handling them (Rodriguez-Morales et al. 2020).

Susceptibility to SARS-CoV-2 in domestic animals, such as pigs and poultry, has not been reported yet following experimental inoculation (Hobbs and Reid 2020; Shi et al. 2020; Mallapaty 2020a). Following the report of SARS-CoV-2 natural infection in farmed minks, a serological survey was conducted among stray cats/feral cats found in the surroundings of mink farms in the Netherlands. Among the twenty-four stray cats screened, seven cats showed positive results for presence of SARS-CoV-2-specific antibodies. However, only one cat tested positive for viral RNA (Oreshkova et al. 2020). The presence of SARS-CoV-2 antibodies in stray cats/feral cats found in the surroundings of mink farms may indicate the possibility of transmission from mink-to-cat rather than human-to-cat since the stray cats/feral cats did not enter the houses of people. The feral cats that were present around mink farms might have acquired the infection during close contact while foraging the farms for food (Csiszar et al. 2020; Enserink 2020).

**Figure 1.**
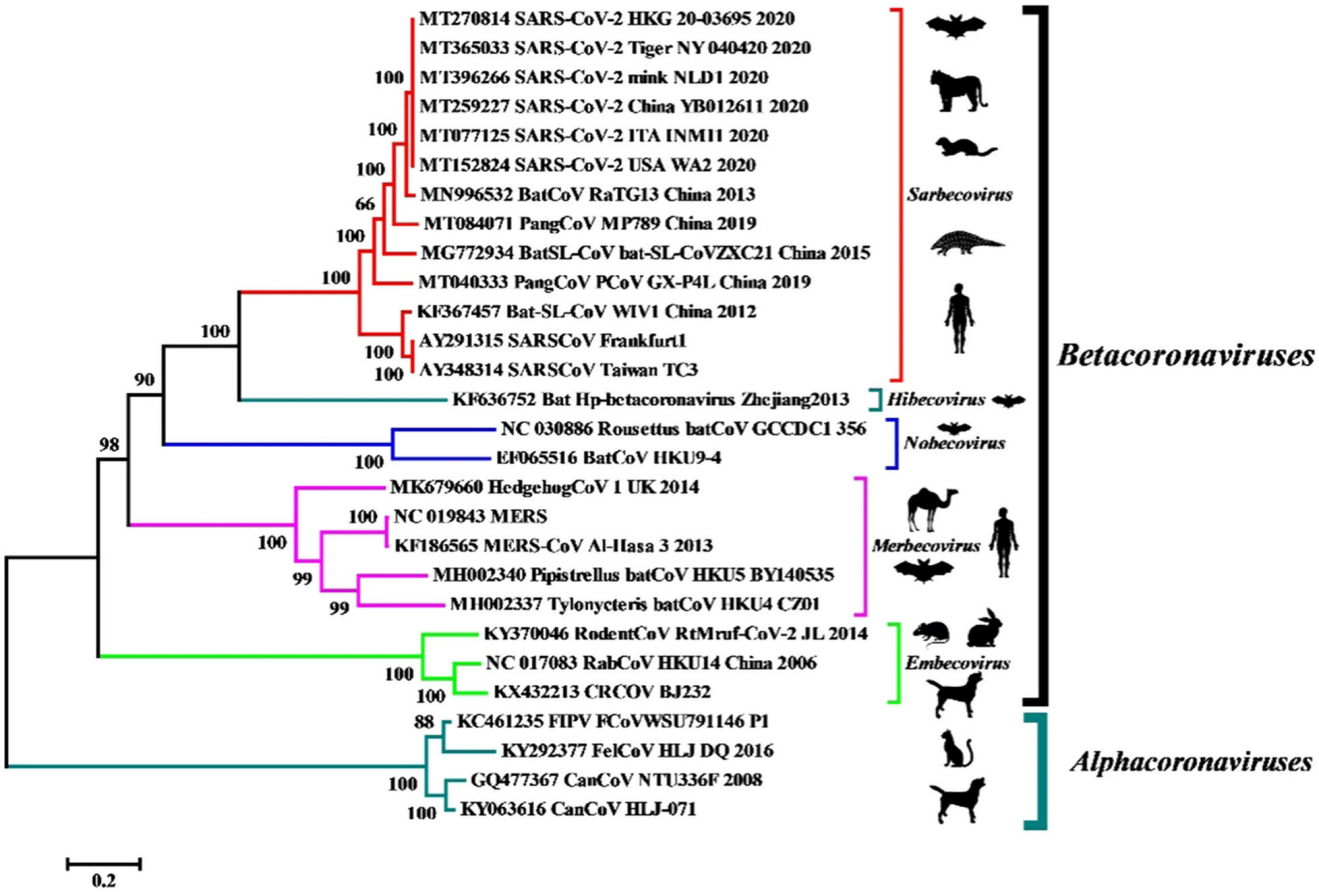
Phylogenetic analysis of human and animal coronaviruses including the SARS-CoV-2 isolated from tiger, mink and human beings. Reproduced from Salajegheh Tazerji et al. (2020) under Creative Commons Attribution (CC BY) licence.

Furthermore, in the event where the SARS-CoV-2 transmission spectrum extends to the wild mustelids (minks and ferrets), the animals can later develop into permanent reservoir hosts and can transmit the infection to human beings and other susceptible animal species (Delahay et al. 2020; Manes et al. 2020). The increasing cases of COVID-19 in humans will further enable elucidation of the mechanisms of transmission of SARS-CoV-2 to susceptible animal species, and hence may result in the establishment of viral reservoirs that can transmit the virus again to human beings (Opriessnig and Huang 2020). The available data provide evidence of SARS‐CoV‐2 transmission from humans to certain animal species within the families *Felinae*, *Caninae*, and *Mustelidae* (Opriessnig and Huang 2020; Oreshkova et al. 2020; Sit et al. 2020). The SARS-CoV-2 isolated from minks and tigers exhibited 99.6%–99.9% homology with the human isolates collected from different parts of the world (Figure 1) (Salajegheh Tazerji et al. 2020).

The genetic sequences of SARS-CoV-2 strains isolated from non-human mammalian species, such as dog, cat, tiger, and mink, were compared to the reference strain. The lack of evident differences and maximum genetic homology among the different strains correspond to the possibility of evolution from a common ancestor (Ul-Rahman et al. 2020). Additionally, the residue substitutions observed at the spike protein-RBD indicate the need for genomic alterations for the successful adaptation to a novel host. SARS-CoV-2 has already been declared a pandemic by the World Health Organization. However, the virus also possesses panzootic potential owing to its broad host range and the inherent ability to cross the ‘species barrier’, thereby highlighting the need for a One Health approach. The establishment of SARS-CoV-2 in any susceptible animal population will enable the recirculation of the adapted animal viruses back into the human population (Gollakner and Capua 2020). This will ensure continuous human infections by SARS-CoV-2 even after successful containment of the primary outbreak.

## 3. Transmission of SARS-CoV-2 to minks

**Figure 2.**
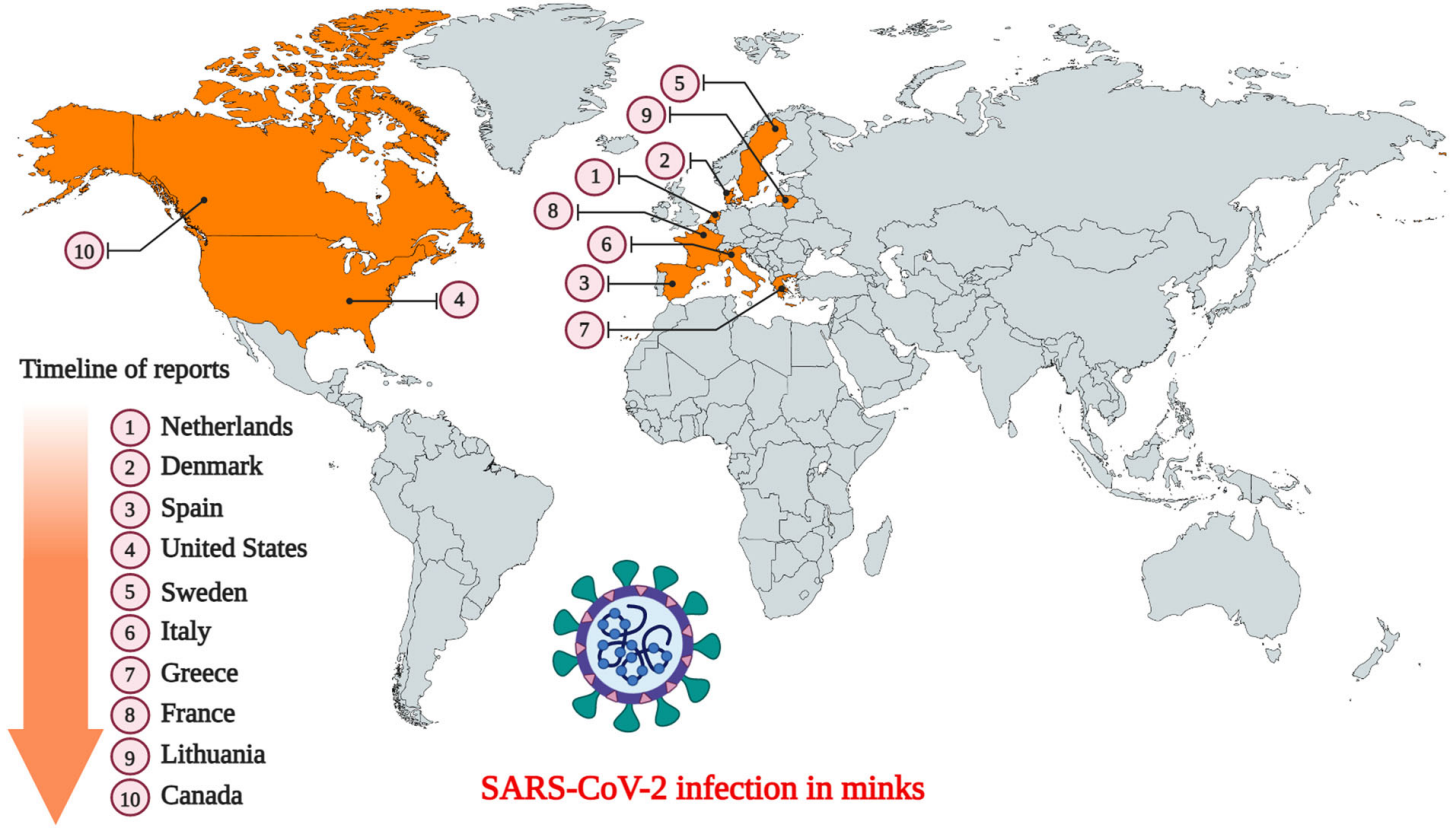
Countries that have reported SARS-CoV-2 infection in farmed minks. (Events in animals, Data retrieved from: https://www.oie.int/en/scientific-expertise/specific-information-and-recommendations/questions-and-answers-on-2019novel-coronavirus/events-in-animals/).

European (*Mustela vison*) and American (*Neovison vison*) minks are members of the weasel family (*Mustelidae*). They are widely farmed in several countries for their fur. SARS-CoV-2 infection in minks was first reported in the Netherlands following separate outbreaks in two farms (Oreshkova et al. 2020). Following the report of widespread mortality in the mink farms of the United States, the U.S. Department of Agriculture confirmed that the minks were infected with the SARS-CoV-2 (Cahan 2020). Currently, SARS-CoV-2 infection in minks has already been documented in ten countries namely the USA, the Netherlands, Sweden, Italy, Denmark, France, Canada, Greece, Lithuania, and Spain (Figure 2) (Cahan 2020; Enserink 2020; OIE 2020; WHO 2020a). Fearing the possibility of minks harboring the virus indefinitely, authorities in these countries have already initiated mass culling to prevent a possible spread to humans (Enserink 2020 Enserink 2020). Postmortem examination of the lungs identified all signs of pneumonia in the infected minks, indicating presence of a respiratory disease (Cahan 2020; Oreshkova et al. 2020). SARS-CoV-2 infection tends to spread rapidly among minks housed in the farms, resulting in mass mortality (Cahan 2020). Since the minks are housed separately in cages with a non-permeable partition between them, animal-to-animal transmission is not possible through direct contact. However, the cages are mostly open wire top cages, so contaminated aerosols/dust can exit and enter the cages through the top. Therefore, it is believed that the virus was transmitted indirectly either through fomites (feed or bedding material), infectious droplets, or contaminated dust from the bedding (fecal matter) (Oreshkova et al. 2020). The presence of SARS-CoV-2 RNA in airborne inhalable dust collected from the mink farms indicates the possibility of indirect transmission between mink-to-mink, and mink-to-human/or other animals (Oreshkova et al. 2020; Zhao et al. 2020a). Therefore, minks can serve as an efficient intermediate host for SARS-CoV-2 in humans (Zhao et al. 2020a).

**Figure 3.**
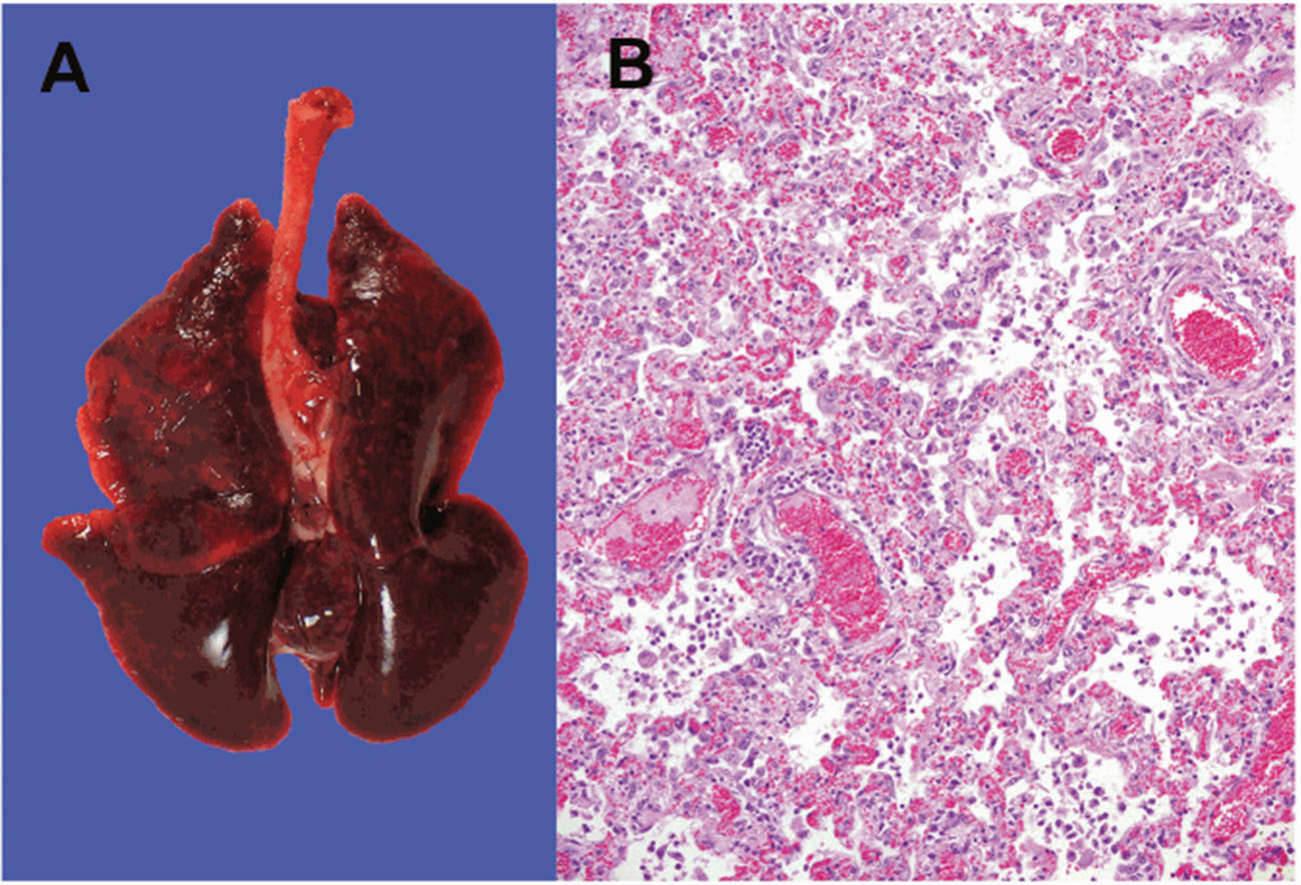
SARS-CoV-2 infection in mink. (A) Macroscopic image of the lungs from a mink infected with SARS-CoV-2. (B) Photomicrograph of a histopathological lung section showing interstitial pneumonia (haematoxylin and eosin, objective 20×). Reproduced from Oreshkova et al. (2020) under Creative Commons Attribution (CC BY) licence.

The necropsies performed on recently dead animals from the mink farms in Netherlands identified diffusely dark to mottled red, wet lung lobes that did not collapse while opening the thoracic cavity (Figure 3A). Histopathological examination of the lung samples collected from animals with evident macroscopic changes in the lungs demonstrated pathological changes suggestive of severe diffuse interstitial pneumonia with hyperemia, alveolar damage, and loss of air-containing alveolar lumina (Figure 3B) (Molenaar et al. 2020; Oreshkova et al. 2020). Acute interstitial pneumonia was the most characteristic postmortem finding reported in nearly all the minks that died during the SARS-CoV-2 outbreak in the Netherlands (Molenaar et al. 2020). Other histologic findings in the lungs include the presence of multifocal to coalescing areas with degeneration and thickening of alveolar septa often lined by delicate hyaline membranes and exhibiting moderate to severe proliferation of type II pneumocytes. The alveolar septa were found to be thickened because of the presence of a fibrillar eosinophilic material. The alveolar lumina were filled with desquamated cells, mononuclear inflammatory cells, and few neutrophils (Molenaar et al. 2020). Other consistent findings included perivascular edema, pulmonary alveolar edema, and hyperemia of alveolar septa. The severity of diffuse alveolar damage varied among the individual minks of different farms.

SARS-CoV-2 RNA was detected in the throat swab, rectal swab, lung samples, and conchae samples collected from the minks. Additionally, RNA was also detected in the liver and intestine, but not in the spleen (Oreshkova et al. 2020). Although the outbreak lasted only for 4 weeks, a proportion of the minks tested PCR-positive for SARS-CoV-2 in the throat swabs even after the resolution of clinical signs (Molenaar et al. 2020). The clinical and pathological findings of SARS-CoV-2 infection and the rapid animal-to-animal transmission in minks are almost similar to the findings observed in patients with COVID-19. Although SARS-CoV-2 infections appear asymptomatic in dogs (Shi et al. 2020; Sit et al. 2020), it manifests as a mild to moderate disease in felines with the involvement of the respiratory and/or gastrointestinal systems (Shi et al. 2020), and as a severe to fatal disease in minks (Cahan 2020; Hobbs and Reid 2020; Oreshkova et al. 2020). Although SARS-CoV-2 infection in minks can induce acute interstitial pneumonia with diffuse alveolar damage, none of the other organ systems were affected. Furthermore, few infected minks show subclinical SARS-CoV-2 infection (Molenaar et al. 2020).

## 4. Evidence for mink-to-human transmission and its implications

Following reports of human-to-mink transmission of SARS-CoV-2, it has been suggested that the virus evolves because of the widespread and rapid circulation among minks (Oude Munnink et al. 2020). Oude Munnink et al. (2020) reported the first animal-to-human SARS-CoV-2 transmission from infected minks to susceptible farmworkers in the Netherlands. Therefore, direct contact with SARS-CoV-2 infected minks should also be considered a risk factor for contracting COVID-19 (Enserink 2020; Oude Munnink et al. 2020). The study also identified high diversity in the SARS-CoV-2 genomic sequences obtained from a few mink farms. This indicated prolonged circulation of the virus among the minks, which might have led to accumulation of mutations that increased virulence of the virus (Oude Munnink et al. 2020).

A total of 214 human cases of COVID-19 have been identified in Denmark with different SARS-CoV-2 variants that is believed to have originated from farmed minks (WHO 2020a). At the time of writing, Statens Serum Institut (State Serum Institute), Denmark, has identified a total of seven unique mutations in the spike protein of SARS-CoV-2 variants found co-circulating in both minks and human beings (WHO 2020b). The clinical presentation and disease severity among those infected with these new variants are similar to those of other circulating SARS-CoV-2 strains. However, a new unique variant referred to as the ‘Cluster 5’, has a series of mutations that are not reported elsewhere. The preliminary results indicated that the new mink-associated variant, identified in both minks and humans, has a moderately reduced sensitivity to neutralizing antibodies (WHO 2020a). However, following detailed analysis, it was confirmed that the mink-associated mutations are not associated with rapid spread, nor with any changes in morbidity and mortality (Mallapaty 2020b). One mink-associated mutation, Y453F, was found to be associated with an amino acid change in the spike protein. The mutation was reported in the SARS-CoV-2 sequences obtained from people in Denmark and the Netherlands (Mallapaty 2020b). This mutant (Y453F) of SARS-CoV-2 is known to have spread widely among the humans (Hayashi et al. 2020b). An experimental study was conducted with this virus variant (Y453F mutation) using three-dimensional protein structural analysis. The findings suggest that the mink-associated mutant can partially escape from getting detected by four commercial neutralizing monoclonal antibodies (mAb) (CC12.1, CC12.3, COVA2-04, and CV07-250) owing to the weak recognition of mAb to spike glycoproteins (Hayashi et al. 2020b; Mallapaty 2020b). Although further studies are necessary to confirm the potential implications of these findings (WHO 2020a), we can assume that the new variant might have a direct impact on the diagnostic techniques, therapeutics, and vaccines that are currently under development. The Cluster 5 variant identified among the infected farm workers has not been reported since September 2020, indicating limited spread within the human population (Mallapaty 2020b). SARS-CoV-2 RNA was detected in the inhalable dust samples collected from the mink farms. The inhalable dust may act as a potential source of infection for the farmworkers (Oreshkova et al. 2020). The animal-to-animal transmission in mink farms is believed to have occurred through contaminated dust from the bedding (fecal matter). Therefore, inhalation of the contaminated dust might have contributed to the transmission of SARS-CoV-2 from minks-to-humans.

**Figure 4.**
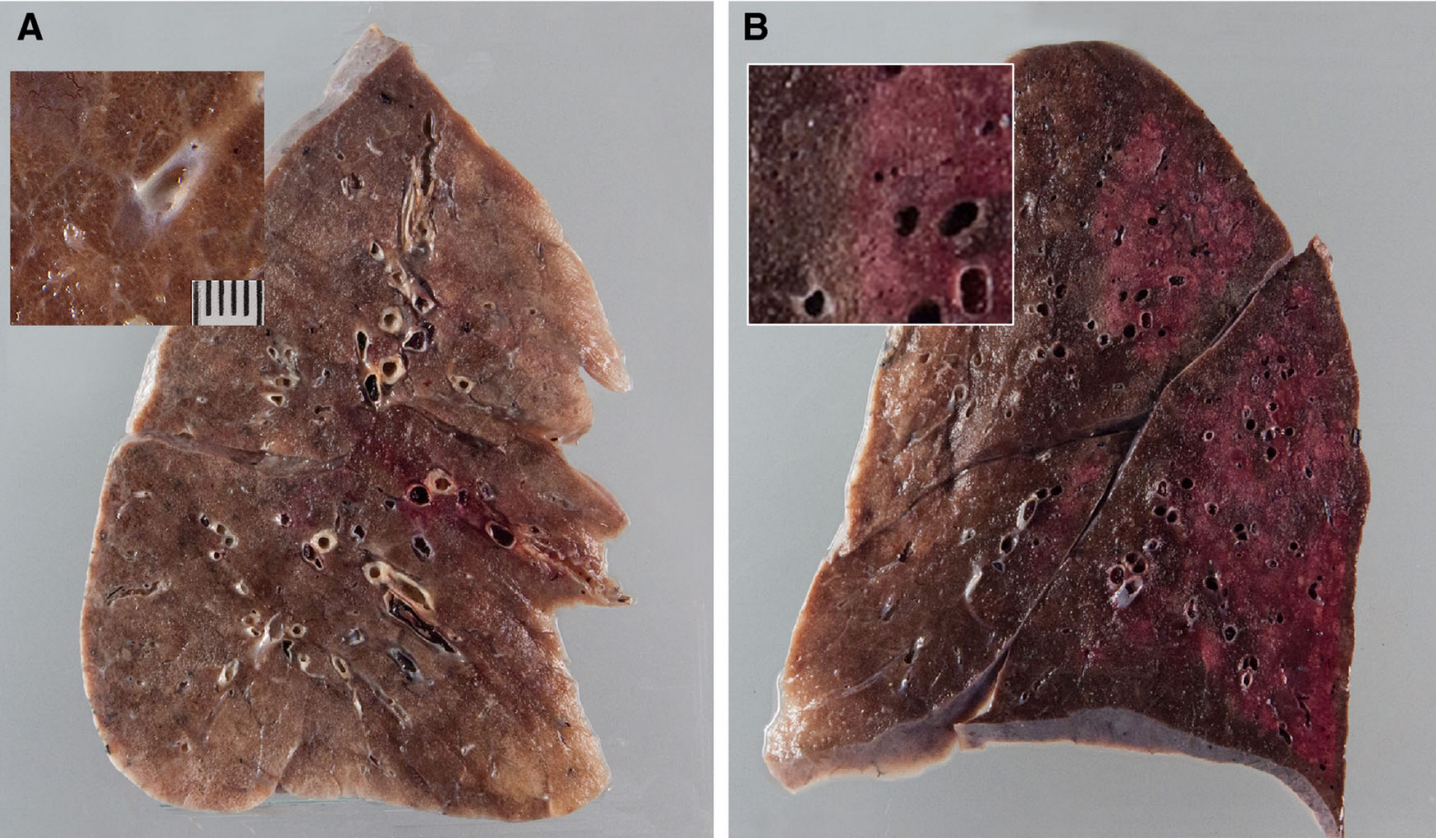
Gross lung findings observed during the postmortem examination of COVID‐19 human patient. (A) Appearance of lungs in a patient with COVID‐19. The thickened alveolar septae and congestive interstitial aspects are also visible in addition to the interstitial congestion (insert). (B) Severe and extensive suppurative bronchopneumonic infiltrates (insert) observed in a patient suffering from COVID‐19. Reproduced with modifications from Menter et al. (2020) under Creative Commons Attribution (CC BY) licence.

**Figure 5.**
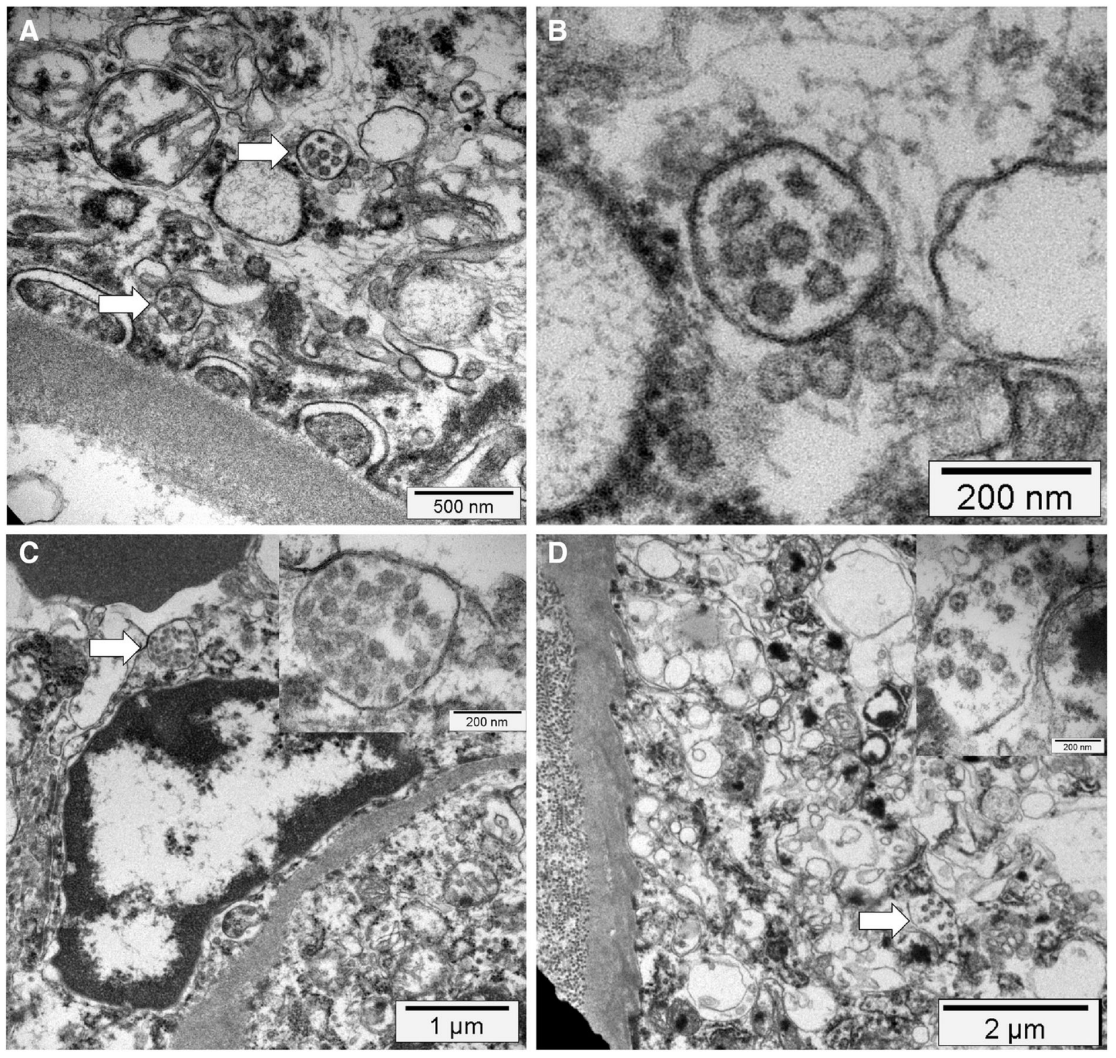
Electron microscopy findings. (A) Transmission electron micrograph of kidney section showing virus-like particles (sizes between 70 nm and 110 nm) within podocyte cytoplasm (arrow). (B) The vesicles contain virus‐like particles with a ring of electron‐dense granules. (C) Virus‐like particles (arrow and insert) within a vesicle that is close to the luminal border. (D) Cytoplasm of a proximal tubular epithelial cell with vesicle containing virus‐like particles (arrow and insert). Reproduced from Menter et al. (2020) under Creative Commons Attribution (CC BY) licence.

SARS-CoV-2 infection in humans might result in severe and fatal respiratory disease with manifestations involving other organ systems. Gross findings observed in the post mortem examination of the lungs of COVID-19 patients include patchy to diffuse areas of consolidation, extensive suppurative bronchopneumonic infiltrates, and firm, heavy, bluish–red colored lung parenchyma with signs of severe congestion (Figure 4) (Menter et al. 2020). The kidneys of patients with COVID-19 showed diffuse acute tubular injury with interstitial edema, widened tubular lumina, and flattened tubular epithelium. Furthermore, transmission electron microscopy identified podocytes with multiple vesicles containing virus-like particles (Figure 5) (Menter et al. 2020). Histopathological examination of the lung samples collected from patients with COVID-19 have demonstrated a broad range of pathological changes including diffuse alveolar damage with disruption of alveolar septa, alveolitis with atrophy, focal interstitial thickening, necrotizing bronchiolitis, metaplasia of alveolar epithelial cells, and bacterial bronchopneumonia (Tian et al. 2020; Yao et al. 2020). Diffuse alveolar damage is the predominant histopathologic lung pathology identified among the patients with COVID-19 (Hariri et al., 2020).

## 5. Mitigation strategies including the one health approach

**Figure 6.**
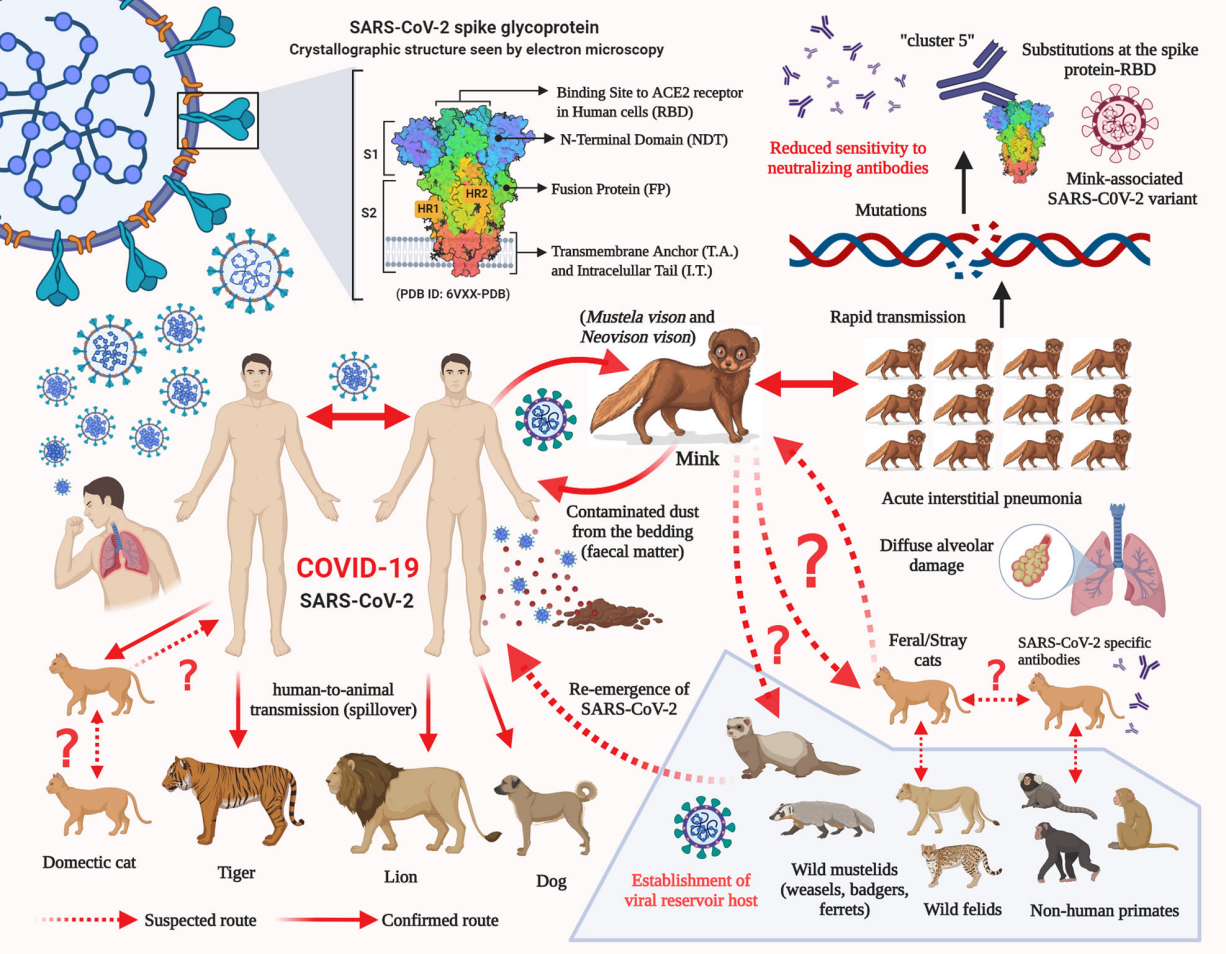
Potential and established routes of SARS-CoV-2 transmission between humans and animal species.

As the human population has increased alarmingly during the last century, the competition for resources and frequent human-animal interactions has become inevitable. Additionally, the economic trade involving wild and exotic animals has also resulted in a closer human-wild animal interaction and it poses a considerable threat via possible transmission of new viruses into the human population. This may lead to the exchange of viruses across species and the emergence of new viral diseases in the human population. Reports on newly emerging diseases in humans, which have originated from animals, during the last few decades have provided evidence of the drawbacks of this interaction. In the current scenario, for most of the emerging health problems and diseases, there is a close relationship/interaction between humans, animals, and their environment. Hence, the emergence of new pathogens may occur at the interface of such interactions and may later lead to the development of a pandemic. However, the emergence of novel pathogens can be prevented by studying the human-animal-ecosystem interface. The One Health approach offers a great strategy to combat the emergence of infectious diseases (Hassani and Khan 2020). From the available data, it is evident that SARS-CoV-2 can adapt to infect a wide range of host species. The emergence of different SARS-CoV-2 variants with unique pathobiological characteristics as a consequence of human-to-animal and animal-to-human transmissions with events of crossing of the species barrier may pose a challenge to the effectiveness of the diagnostic techniques, therapeutics, and vaccines that are currently under development to counter the COVID-19 pandemic. Adaptation and evolution of the SARS-CoV-2 are being reported and has been attributed to the increased genetic diversity among SARS-CoV-2-infected patients (Shen et al. 2020). The transmission of SARS-CoV-2 from humans to minks has already resulted in the emergence of a new mink-associated virus variant (Figure 6).

The detection of SARS-CoV-2 viral RNA in the airborne inhalable dust (contaminated with fecal matter) collected from the mink farms indicates the possibility of animal-to-animal and animal-to-human transmission of SARS-CoV-2 via dust and/or droplets. Therefore, the workers/employees are subjected to the risk of SARS-CoV-2 exposure while working on mink farms. In addition to the countries that have reported SARS-CoV-2 infection in minks, namely the USA, the Netherlands, Sweden, Italy, Denmark, France, Canada, Greece, Lithuania, and Spain, mink farms are spread across several other countries in Europe. Therefore, considering the potential for virus mutation events in minks and evidence of mink-to-human transmission, strict biosecurity measures should be implemented to prevent the further transmission of the mink-associated SARS-CoV-2 variant to human beings. This can be made possible by increasing awareness among the scientific community and the employees/workers of the mink industry that similar to humans, minks are highly susceptible to SARS-CoV-2 infection and can pose high public health concerns via the additional zoonotic transmission of COVID-19 amidst the ongoing pandemic. The rapid human-to-human transmission is presently at its highest with the increasing number of SARS-CoV-2 confirmed cases, leading to higher global cumulative mortality. It is increasingly difficult to control the accelerated transmission of the disease owing to the unavailability of any effective drug and/or vaccine to combat this pandemic. Further studies and surveillance are warranted to determine and confirm the role of the different animal species in the zoonotic and community transmission of SARS-CoV-2 and to design appropriate strategies to combat these situations.

The surveillance and monitoring of SARS-CoV-2 in animals including livestock, companion, pet, and zoo animals and their handlers as well as other wildlife species should be strengthened along with the implementation of a wider One Health approach, mitigation strategies, and preventive and control strategies to contain this virus as well as its zoonosis, the mechanisms of which remains unclear (Dhama et al. 2013; Bhatia 2020; Leroy et al. 2020; McNamara et al. 2020; Murdoch and French 2020). Apart from this, the research and development activities involved in investigation of SARS-CoV-2 infection in animals and their susceptibility should be conducted with greater emphasis across the globe, which would aid in the design and modification of appropriate prevention and control strategies to limit the transmission of this virus as well as help to tackle future similar pandemics and epidemics of infectious diseases, especially those emerging at animal-human interfaces.

While the entire world is grappled with the ongoing COVID-19 pandemic caused by SARS-CoV-2 and as relentless efforts are being directed toward the development of a potent and successful vaccine for the prevention and control of the virus, along with extensive studies on targeted antiviral drugs for treating patients, the emergence of any new variant virus may derail the progress made thus far to contain the virus and its transmission. The variant viruses that can escape the virus-induced immunity or neutralizing effect of monoclonal antibodies and the inhibitory effect of antiviral drugs developed against the wild type virus, may render the newly developed vaccine or antiviral drug ineffective. Considering the substantial impact of the newly emerged variant virus strain of SARS-CoV-2 on public health and the world economy, the approach of culling all animals involved in the emergence and transmission of the variant virus may be considered as a last resort even though this may seem unethical as well. This approach has been considered in the past during many disease outbreaks such as the bird flu in different countries. However, this approach is highly effective if it is implemented during the early stage of transmission of the new variant virus when it is restricted to a specific animal and human population in a farm or country. It may not be effective during the late stage of transmission when the new variant is already transmitted and spread to considerable human and animal populations across different geographical locations. It is also effective if the variant only circulates in the farmed minks. Transmission to wild minks may pose a further challenge in elimination of the variant virus. As the variant SARS-CoV-2 virus strain has been already detected in various countries across different continents, it appears to have already spread across a large geographical region. The success of preventing the spread of this variant strain depends on the coordinated international efforts involving all the affected countries. Isolated efforts may not yield the desired result to stop the global spread of the variant virus. International efforts should include the ethical and economic aspects of the control strategy by culling all minks and by involving all the stakeholders, and it should be governed by acceptable international laws.

## 6. Conclusion and future prospects

Since the emergence of the COVID-19 pandemic, normal human life and economic activities have been severely affected globally. The return to normalcy depends on the extent of progress made in the diagnostic technique, therapeutic, and vaccine design aspects to control the pandemic, which can be sustained by preventing the emergence of any new variant SARS-CoV-2 strains. Scientific evidence-based control strategies should be adopted to stop the spread of a new variant strain at the early stage of identification when it is restricted to a population of humans or animals. Surveillance and monitoring of the evolution of new variant viruses should be carried out routinely in human and animal populations by considering the One Health approach. In the event of the identification of a new variant, coordinated international efforts to contain the spread based on the One Health approach should be undertaken. The animal-human interaction, particularly, with wild animals, such as minks, pangolins, bats, and ferrets should be avoided. The food habits or economic activities involving these animals should be prevented to block/control any possible transmission of pathogens from one of these species to humans in the future. Awareness of the One Health approach is warranted through training and education for all the stakeholders involved in human, animal, and environmental health policies to address the effective control of newly emerging diseases such as COVID-19.

Since its emergence, the SARS-CoV-2 continues to accumulate mutations and evolve progressively. However, the emergence of a new variant in humans highlight the role played by minks in accelerating the process of evolution. The effects of this new SARS-CoV-2 variant remain unclear at this juncture. However, such virus variants may pose a threat to the efficacy of monoclonal antibodies and vaccines that are currently under development and clinical trials. It is evident that the mink farms will further play a major role in the emergence of newer and more virulent variants of SARS-CoV-2 that may not respond to our therapeutic strategies. The mink farms house thousands of minks in close proximity, which is an ideal condition for promotion of the rapid transmission of SARS-CoV-2 under a closed environment. The rapid spread of SARS-CoV-2 across the mink farms in Europe and North America calls for an immediate response from the concerned authorities to devise new strategies to prevent further spread. Since new SARS-CoV-2 variants can emerge from mink farms as a consequence of human-to-mink and mink-to-human transmission, preventive measures should be established to block the interaction between the two highly susceptible species. Furthermore, it is not clear whether SARS-CoV-2 can be transmitted to other animals such as feral/stray cats living in close proximity to the infected mink farms. Therefore, further studies should be conducted to screen for feral/stray animals living around the farms and to investigate their role in promoting cross-species transmission of SARS-CoV-2.

## References

[CIT0001] Bhatia R. 2020. Need for integrated surveillance at human-animal interface for rapid detection & response to emerging coronavirus infections using one health approach. Indian J Med Res. 151(2 & 3):132–135. 32202259 10.4103/ijmr.IJMR_623_20 PMC7357400

[CIT0002] Cahan E. 2020. COVID-19 hits US mink farms after ripping through Europe. Science. 80.

[CIT0003] Csiszar A, Jakab F, Valencak TG, Lanszki Z, Tóth GE, Kemenesi G, Tarantini S, Fazekas-Pongor V, Ungvari Z. 2020. Companion animals likely do not spread COVID-19 but may get infected themselves. Geroscience. 42(5):1229–1236. 32766998 10.1007/s11357-020-00248-3 PMC7410515

[CIT0004] Delahay R, de la Fuente J, Smith G, Sharun K, Snary E, Flores Giron L, Nziza J, Fooks A, Brookes S, Lean F, et al. 2020. Assessing the risks of SARS-CoV-2 in wildlife. Preprints. 2020120283. doi: 10.20944/preprints202012.0283.v1 PMC8024038 33834160

[CIT0005] Deng W, Bao L, Gao H, Xiang Z, Qu Y, Song Z, Gong S, Liu J, Liu J, Yu P, et al. 2020. Ocular conjunctival inoculation of SARS-CoV-2 can cause mild COVID-19 in rhesus macaques. Nat Commun. 11(1):4400. 32879306 10.1038/s41467-020-18149-6 PMC7467924

[CIT0006] Dhama K, Khan S, Tiwari R, Sircar S, Bhat S, Malik YS, Singh KP, Chaicumpa W, Bonilla-Aldana DK, Rodriguez-Morales AJ. 2020a. Coronavirus disease 2019-COVID-19. Clin Microbiol Rev. 33(4):e00028-20. 32580969 10.1128/CMR.00028-20 PMC7405836

[CIT0007] Dhama K, Patel SK, Sharun K, Pathak M, Tiwari R, Yatoo MI, Malik YS, Sah R, Rabaan AA, Panwar PK, et al. 2020b. SARS-CoV-2 jumping the species barrier: zoonotic lessons from SARS, MERS and recent advances to combat this pandemic virus. Travel Med Infect Dis. 37:101830. 32755673 10.1016/j.tmaid.2020.101830 PMC7396141

[CIT0008] Dhama K, Verma AK, Tiwari R, Chakraborty S, Vora K, Kapoor S, Deb R, Karthik K, Singh R, Munir M, et al. 2013. A perspective on applications of geographical information system (GIS); an advanced tracking tool for disease surveillance and monitoring in veterinary epidemiology. Adv Anim Vet Sci. 1(1):14–24.

[CIT0009] Enserink M. 2020. Coronavirus rips through Dutch mink farms, triggering culls. Science. 368(6496):1169. 32527808 10.1126/science.368.6496.1169

[CIT0010] Gollakner R, Capua I. 2020. Is COVID-19 the first pandemic that evolves into a panzootic? Vet Ital. 56(1):7–8. 32315124 10.12834/VetIt.2246.12523.1

[CIT0011] Hariri LP, North CM, Shih AR, Israel RA, Maley JH, Villalba JA, Vinarsky V, Rubin J, Okin DA, Sclafani A, et al. 2020. Lung histopathology in Coronavirus disease 2019 as compared with severe acute respiratory sydrome and H1N1 influenza: a systematic review. Chest. Epub ahead of print. PMID: 33038391; PMCID: PMC7538870. 10.1016/j.chest.2020.09.259 PMC7538870 33038391

[CIT0012] Hassani A, Khan G. 2020. Human-animal interaction and the emergence of SARS-CoV-2. JMIR Public Health Surveill. 6(4):e22117. 33001837 10.2196/22117 PMC7546868

[CIT0013] Hayashi T, Abiko K, Mandai M, Yaegashi N, Konishi I. 2020a. Highly conserved binding region of ACE2 as a receptor for SARS-CoV-2 between humans and mammals. Vet Q. 40(1):243–249. 32921279 10.1080/01652176.2020.1823522 PMC7580767

[CIT0014] Hayashi T, Yaegashi N, Konishi I. 2020b. Effect of RBD mutation (Y453F) in spike glycoprotein of SARS-CoV-2 on neutralizing antibody affinity. bioRxiv. 37805871

[CIT0015] Hemida MG, Elmoslemany A, Al-Hizab F, Alnaeem A, Almathen F, Faye B, Chu DK, Perera RA, Peiris M. 2017. dromedary camels and the transmission of Middle East Respiratory Syndrome Coronavirus (MERS-CoV). Transbound Emerg Dis. 64(2):344–353. 26256102 10.1111/tbed.12401 PMC4749478

[CIT0016] Hobbs EC, Reid TJ. 2020. Animals and SARS-CoV-2: species susceptibility and viral transmission in experimental and natural conditions, and the potential implications for community transmission. Transbound Emerg Dis. 10.1111/tbed.13885 PMC8359434 33091230

[CIT0017] Jo WK, de Oliveira-Filho EF, Rasche A, Greenwood AD, Osterrieder K, Drexler JF. 2020. Potential zoonotic sources of SARS-CoV-2 infections. Transbound Emerg Dis. 10.1111/tbed.13872 PMC7675418 33034151

[CIT0018] Leroy EM, Ar Gouilh M, Brugère-Picoux J. 2020. The risk of SARS-CoV-2 transmission to pets and other wild and domestic animals strongly mandates a one-health strategy to control the COVID-19 pandemic. One Health. 10:100133. 32363229 10.1016/j.onehlt.2020.100133 PMC7194722

[CIT0019] Low-Gan J, Huang R, Warner G, Kelley A, McGregor D, Smider V. 2020. Diversity of ACE2 and its interaction with SARS-CoV-2 receptor binding domain. bioRxiv [Preprint]. 10.1042/BCJ20200908 34558627

[CIT0020] Lu S, Zhao Y, Yu W, Yang Y, Gao J, Wang J, Kuang D, Yang M, Yang J, Ma C, et al. 2020. Comparison of nonhuman primates identified the suitable model for COVID-19. Signal Transduct Target Ther. 5(1):157. 32814760 10.1038/s41392-020-00269-6 PMC7434851

[CIT0021] Malik YS, Sircar S, Bhat S, Sharun K, Dhama K, Dadar M, Tiwari R, Chaicumpa W. 2020. Emerging novel coronavirus (2019-nCoV)-current scenario, evolutionary perspective based on genome analysis and recent developments. Vet Q. 40(1):68–76. 32036774 10.1080/01652176.2020.1727993 PMC7054940

[CIT0022] Mallapaty S. 2020a. Coronavirus can infect cats - dogs, not so much. Nature. 10.1038/d41586-020-00984-8 32238897

[CIT0023] Mallapaty S. 2020b. COVID mink analysis shows mutations are not dangerous - yet. Nature. 587(7834):340–341. 33188367 10.1038/d41586-020-03218-z

[CIT0024] Manes C, Gollakner R, Capua I. 2020. Could Mustelids spur COVID-19 into a panzootic? Vet Ital. 10.12834/VetIt.2375.13627.1 32909703

[CIT0025] McNamara T, Richt JA, Glickman L. 2020. A critical needs assessment for research in companion animals and livestock following the pandemic of COVID-19 in humans. Vector Borne Zoonotic Dis. 20(6):393–405. 32374208 10.1089/vbz.2020.2650 PMC7249469

[CIT0026] Menter T, Haslbauer JD, Nienhold R, Savic S, Hopfer H, Deigendesch N, Frank S, Turek D, Willi N, Pargger H, et al. 2020. Postmortem examination of COVID-19 patients reveals diffuse alveolar damage with severe capillary congestion and variegated findings in lungs and other organs suggesting vascular dysfunction. Histopathology. 77(2):198–209. 32364264 10.1111/his.14134 PMC7496150

[CIT0027] Molenaar RJ, Vreman S, Hakze-van der Honing RW, Zwart R, de Rond J, Weesendorp E, Smit LAM, Koopmans M, Bouwstra R, Stegeman A, et al. 2020. Clinical and pathological findings in SARS-CoV-2 disease outbreaks in farmed mink (Neovison vison). Vet Pathol. 57(5):653–657. 32663073 10.1177/0300985820943535

[CIT0028] Muñoz-Fontela C, Dowling WE, Funnell SGP, Gsell PS, Balta XR, Albrecht RA, Andersen H, Baric RS, Carroll MW, Cavaleri M, et al. 2020. Animal models for COVID-19. Nature. 586(7830):509–515. 32967005 10.1038/s41586-020-2787-6 PMC8136862

[CIT0029] Murdoch DR, French NP. 2020. COVID-19: another infectious disease emerging at animal-human interface. New Zealand Med J. 133(1510):12–15. 32078596

[CIT0030] Mykytyn AZ, Lamers MM, Okba NM, Breugem TI, Schipper D, van den Doel PB, van Run P, van Amerongen G, de Waal L, Koopmans M, et al. 2020. Susceptibility of rabbits to SARS-CoV-2. bioRxiv. 10.1080/22221751.2020.1868951 PMC7832544 33356979

[CIT0031] OIE. 2020. Events in animals. [accessed 2020 Dec 16]. https://www.oie.int/en/scientific-expertise/specific-information-and-recommendations/questions-and-answers-on-2019novel-coronavirus/events-in-animals/.

[CIT0032] Opriessnig T, Huang YW. 2020. Further information on possible animal sources for human COVID-19. Xenotransplantation. 27(6):e12651. 32978828 10.1111/xen.12651 PMC7536993

[CIT0033] Oreshkova N, Molenaar RJ, Vreman S, Harders F, Oude Munnink BB, Hakze-van der Honing RW, Gerhards N, Tolsma P, Bouwstra R, Sikkema RS, et al. 2020. SARS-CoV-2 infection in farmed minks, the Netherlands, April and May 2020. Euro Surveill. 25(23):2001005. 10.2807/1560-7917.ES.2020.25.23.2001005 PMC7403642 32553059

[CIT0034] Oude Munnink BB, Sikkema RS, Nieuwenhuijse DF, Molenaar RJ, Munger E, Molenkamp R, van der Spek A, Tolsma P, Rietveld A, Brouwer M, et al. 2020. Transmission of SARS-CoV-2 on mink farms between humans and mink and back to humans. Science. eabe5901. Nov 10:10.1126/science.abe5901 PMC7857398 33172935

[CIT0035] Rockx B, Kuiken T, Herfst S, Bestebroer T, Lamers MM, Oude Munnink BB, de Meulder D, van Amerongen G, van den Brand J, Okba NMA, et al. 2020. Comparative pathogenesis of COVID-19, MERS, and SARS in a nonhuman primate model. Science. 368(6494):1012–1015. 32303590 10.1126/science.abb7314 PMC7164679

[CIT0036] Rodriguez-Morales AJ, Dhama K, Sharun K, Tiwari R, Bonilla-Aldana DK. 2020. Susceptibility of felids to coronaviruses. Vet Rec. 186(17):e21. 10.1136/vr.m1671 32393590

[CIT0037] Salajegheh Tazerji S, Magalhães Duarte P, Rahimi P, Shahabinejad F, Dhakal S, Singh Malik Y, Shehata AA, Lama J, Klein J, Safdar M, et al. 2020. Transmission of severe acute respiratory syndrome coronavirus 2 (SARS-CoV-2) to animals: an updated review. J Transl Med. 18(1):358. 32957995 10.1186/s12967-020-02534-2 PMC7503431

[CIT0038] Schlottau K, Rissmann M, Graaf A, Schön J, Sehl J, Wylezich C, Höper D, Mettenleiter TC, Balkema-Buschmann A, Harder T, et al. 2020. SARS-CoV-2 in fruit bats, ferrets, pigs, and chickens: an experimental transmission study. Lancet Microbe. 1(5):e218–e225. 32838346 10.1016/S2666-5247(20)30089-6 PMC7340389

[CIT0039] Sharun K, Sircar S, Malik YS, Singh RK, Dhama K. 2020a. How close is SARS-CoV-2 to canine and feline coronaviruses? J Small Anim Pract. 61(8):523–526. 32785948 10.1111/jsap.13207 PMC7436317

[CIT0040] Sharun K, Tiwari R, Patel SK, Karthik K, Iqbal Yatoo M, Malik YS, Singh KP, Panwar PK, Harapan H, Singh RK, et al. 2020b. Coronavirus disease 2019 (COVID-19) in domestic animals and wildlife: advances and prospects in the development of animal models for vaccine and therapeutic research. Hum Vaccin Immunother. 1–12. 32915100 10.1080/21645515.2020.1807802 PMC8641595

[CIT0041] Shen Z, Xiao Y, Kang L, Ma W, Shi L, Zhang L, Zhou Z, Yang J, Zhong J, Yang D, et al. 2020. Genomic diversity of severe acute respiratory syndrome-Coronavirus 2 in patients with Coronavirus Disease 2019. Clin Infect Dis. 71(15):713–720. 32129843 10.1093/cid/ciaa203 PMC7108196

[CIT0042] Shi J, Wen Z, Zhong G, Yang H, Wang C, Huang B, Liu R, He X, Shuai L, Sun Z, et al. 2020. Susceptibility of ferrets, cats, dogs, and other domesticated animals to SARS-coronavirus 2. Science. 368(6494):1016–1020. 32269068 10.1126/science.abb7015 PMC7164390

[CIT0043] Sia SF, Yan LM, Chin AWH, Fung K, Choy KT, Wong AYL, Kaewpreedee P, Perera RAPM, Poon LLM, Nicholls JM, et al. 2020. Pathogenesis and transmission of SARS-CoV-2 in golden hamsters. Nature. 583(7818):834–838. 32408338 10.1038/s41586-020-2342-5 PMC7394720

[CIT0044] Sit THC, Brackman CJ, Ip SM, Tam KWS, Law PYT, To EMW, Yu VYT, Sims LD, Tsang DNC, Chu DKW, et al. 2020. Infection of dogs with SARS-CoV-2. Nature. 586(7831):776–778. 32408337 10.1038/s41586-020-2334-5 PMC7606701

[CIT0045] Tian S, Xiong Y, Liu H, Niu L, Guo J, Liao M, Xiao SY. 2020. Pathological study of the 2019 novel coronavirus disease (COVID-19) through postmortem core biopsies. Mod Pathol. 33(6):1007–1014. 32291399 10.1038/s41379-020-0536-x PMC7156231

[CIT0046] Tiwari R, Dhama K, Sharun K, Iqbal Yatoo M, Malik YS, Singh R, Michalak I, Sah R, Bonilla-Aldana DK, Rodriguez-Morales AJ. 2020. COVID-19: animals, veterinary and zoonotic links. Vet Q. 40(1):169–182. 32393111 10.1080/01652176.2020.1766725 PMC7755411

[CIT0047] Ul-Rahman A, Shabbir MAB, Aziz MW, Yaqub S, Mehmood A, Raza MA, Shabbir MZ. 2020. A comparative phylogenomic analysis of SARS-CoV-2 strains reported from non-human mammalian species and environmental samples. Mol Biol Rep. 47(11):9207–9211. 33104993 10.1007/s11033-020-05879-5 PMC7586201

[CIT0048] Ulrich L, Wernike K, Hoffmann D, Mettenleiter TC, Beer M. 2020. Experimental infection of cattle with SARS-CoV-2. Emerg Infect Dis. 26(12): 2979–2981. 33034284 10.3201/eid2612.203799 PMC7706945

[CIT0049] Wang LF, Eaton BT. 2007. Bats, civets and the emergence of SARS. Curr Top Microbiol Immunol. 315:325–344. 17848070 10.1007/978-3-540-70962-6_13 PMC7120088

[CIT0050] WHO. 2020a. SARS-CoV-2 mink-associated variant strain – Denmark (November 6, 2020). [accessed 2020 Nov 8]. https://www.who.int/csr/don/06-november-2020-mink-associated-sars-cov2-denmark/en/.

[CIT0051] WHO. 2020b. SARS-CoV-2 mink-associated variant strain – Denmark (December 3, 2020). [accessed 2020 Dec 16]. https://www.who.int/csr/don/03-december-2020-mink-associated-sars-cov2-denmark/en/.

[CIT0052] Woolsey C, Borisevich V, Prasad AN, Agans KN, Deer DJ, Dobias NS, Heymann JC, Foster SL, Levine CB, Medina L, et al. 2020. Establishment of an African green monkey model for COVID-19. bioRxiv [Preprint]. 10.1038/s41590-020-00835-8 PMC7790436 33235385

[CIT0053] Yao XH, He ZC, Li TY, Zhang HR, Wang Y, Mou H, Guo Q, Yu SC, Ding Y, Liu X, et al. 2020. Pathological evidence for residual SARS-CoV-2 in pulmonary tissues of a ready-for-discharge patient. Cell Res. 30(6):541–543. 32346074 10.1038/s41422-020-0318-5 PMC7186763

[CIT0054] Zhao J, Cui W, Tian BP. 2020a. The Potential intermediate hosts for SARS-CoV-2. Front Microbiol. 11:580137. 33101254 10.3389/fmicb.2020.580137 PMC7554366

[CIT0055] Zhao Y, Wang J, Kuang D, Xu J, Yang M, Ma C, Zhao S, Li J, Long H, Ding K, et al. 2020b. Susceptibility of tree shrew to SARS-CoV-2 infection. Sci Rep. 10(1):16007. 32994418 10.1038/s41598-020-72563-w PMC7525503

